# Health-related Quality of Life in Children and Adolescents With Sagittal Synostosis

**DOI:** 10.1097/SCS.0000000000009733

**Published:** 2023-09-08

**Authors:** Melissa S.I.C Kurniawan, Stephanie D.C. van de Beeten, Hein Raat, Irene M. J. Mathijssen, Clemens M.F. Dirven, Marie-Lise C. van Veelen

**Affiliations:** * Department of Plastic and Reconstructive Surgery and Hand Surgery; † Department of Neurosurgery; ‡ Department of Public Health, Erasmus University Medical Center Rotterdam, The Netherlands

**Keywords:** Craniosynostosis, Headache, Quality of Life, Sagittal synostosis

## Abstract

**Background:**

This study evaluated the health-related quality of life (HR-QoL) in patients with sagittal synostosis (SS), and the influence of frequent headaches and surgical techniques on the HR-QoL.

**Method:**

Patients with SS aged 8 to 18 years were invited to participate between June 2016 and February 2017. The Child Health Questionnaire was used to assess the HR-QoL. A detailed questionnaire was used to assess the severity of headache symptoms. The control group consisted of 353 school children aged 5 to 14 years.

**Results:**

In all, 95 parents of patients with SS were invited to participate, of whom 68 (71.6%) parents completed the CHQ-PF50. The mean age of the participating patients was 12.4 years (10.8 to 14.2). The Psychosocial- and Physical summary of the patients with SS was similar to the general population. In the distinct CHQ scales, “Family cohesion” (*P*=0.02) was higher, and “Mental health” (*P*=0.05) was lower compared with the general population. The type and timing of surgery did not affect the HR-QoL. Thirty-two patients (47.1%) reported having headache complaints at least once a month. The CHQ scores of SS patients with frequent headaches had a significantly lower score of mild to large effect than those without headaches.

**Conclusion:**

Patients with SS have a slightly lower to similar HR-QoL compared with the general population. In all, 47.1% of SS patients have frequent headaches, resulting in lower average HR-QoL. The type and timing of surgery did not affect the results. Clinicians should be aware of lower HR-QoL in some subgroups of patients with SS.

Sagittal synostosis (SS) is a condition in which the sagittal suture fuses prematurely. This leads to an increase in the anteroposterior diameter of the cranium and a decrease in bitemporal diameter. Children with SS are at risk of developing intracranial hypertension (ICH),^1^ which could be caused by a too small intracranial volume^2^ Surgical intervention is crucial since the spontaneous improvement of the skull shape is not expected.^1^

Despite numerous types of surgical interventions being described, there is still no consensus on the optimal approach. Objective measurements, such as cranial index, cranial volume, head circumference, symptoms of ICH, shape analysis, and complications, are used to compare these surgical techniques and can create medical evidence favorable to a certain surgical procedure or technique.^2–5^ However, objective metrics do not consider the potential psychosocial impact.

In recent years, the health-related quality of life (HR-QoL) has become increasingly relevant. Children with SS may have an elevated risk for psychosocial difficulties and lower HR-QoL.^6^ However, the relationship between health and quality of life is complex since a disability does not always imply a negative relationship with quality of life.^7^ Therefore, a superior surgical technique based on objective measurements does not automatically imply a higher HR-QoL in the long term.^8,9^ The information on the HR-QoL in sagittal synostosis patients is still limited.

In addition, headache symptoms are crucial to monitor during follow-up since these may be a clinical sign of ICH and significantly impact the HR-QoL.^10^ Headache complaints are frequent among patients with sagittal synostosis, with our center’s previous study reporting that 38% of patients experience headache complaints regularly.^10^ These complaints have been linked to a smaller head circumference and the use of an Extended Strip Craniotomy (ESC) as a surgical correction technique.^10^ Therefore, a detailed headache assessment is important. Not only to understand the impact frequent headache has on the well-being of patients with sagittal synostosis but also to evaluate the headache characteristics to define and diagnose the type of headache (eg, migraine, tension-type headache, etc.) and its relation to the type of surgery.

This study aims to evaluate the HR-QoL in patients with sagittal synostosis who are previously treated and reported by their parents and compare them with a cohort of unaffected children. The secondary aim is to compare the HR-QoL in SS children with different surgical techniques. In addition, a detailed questionnaire regarding headache complaints and their relation to the CHQ in sagittal synostosis patients is evaluated.

## METHODS

### Study Population

This study was conducted at the Sophia Children’s Hospital, University Medical Center, Rotterdam, The Netherlands. All parents of patients with sagittal synostosis between 8 and 18 years old were invited to participate in this study between June 2016 and February 2017. Patients were excluded from the study if they had syndromic sagittal synostosis or/and if parents/guardians were unable to read or write in Dutch. Parents were asked to complete the CHQ-PF50 questionnaire and headache questionnaire. No rewards or incentives for the completion of the questionnaire were offered. The study was approved by the Medical Ethics Committee (MEC-2014-445).

### Child Health Questionnaire

The Child Questionnaire (CHQ PF50) is parent-reported and was used to evaluate the HR-QoL. The CHQ PF50 was translated into Dutch in 2001 according to international guidelines and has been validated.^11^ The CHQ consists of 50 items across 11 domains and 2 single-item questions. The domains consist of 3 to 6 items with 4, 5, or 6 possible responses per item. Scores were calculated by recoding and summarizing the domain item scores, with a final score ranging from 0 (worst possible health state) to 100 (best possible health state). Two scales (“Physical Summary” and “Psychosocial Summary”) were computed using a factor-analytical model, similar to the summary scores constructed in the SF-36^12^ to derive overall physical and psychosocial scores. These summary scales are based on CHQ scales evolving around the child’s HR-QoL and not scales about family impact. For “Physical Summary” and “Psychosocial Summary”, a mean of 50 represents the general population, with a SD of 10 in either direction.^12,13^

### Additional Headache Questionnaire

To assess information regarding headache symptoms, a detailed questionnaire was composed. The headache questionnaire consists of 12 questions: 6 binary questions and 6 multi-item scale questions to assess the severity, frequency, and type of headache. Six of the questions regarding headaches were based on the PROXY PROMIS. The comprehensive questionnaire was developed to evaluate headache symptoms by a team of experts (eg, a pediatric neurosurgeon (M.vV.), pediatric neurologist (C.C. & J.B.), and public health professional (H.R.) with the help of guidelines of the Dutch College of General Practitioners (NHG-standaard).^14^ The questions are designed to diagnose headaches according to the ICHD criteria (The International Classification of Headache Disorders). To measure the effect of primary headache, a sensitivity analysis of the subgroup was performed.

### Normative Population

To compare our population with the average Dutch population, we utilized data from Raat et al (2002).^11^ This data was collected from 353 school children, aged 5 to 14 years, from 3 elementary schools in Rotterdam, the Netherlands. The CHQ PF50 was used, and parents were asked to complete the questionnaire and return them within 2 weeks. Parents needed to be able to read and write in Dutch to be able to understand and complete the questionnaire. For comparison with children with frequent headaches, we used data from Raat et al.^15^

### Statistical Analysis

Patient characteristics are presented with counts and percentages (for categorical variables), means and SDs (for approximately normally distributed variables), or with Median and IQR (for non-normally distributed variables). CHQ domains are presented using the final score (range of 0 to 100).

The comparison between the study population and the average Dutch population was analyzed using a 2-sample *t*-test, with effect size defined by Cohen’s *d*.^16^ Within the study population, differences were tested using the Mann-Whitney test and the effect size in *r*. The effect size was defined as; <0.3 = small effect, 0.3 –0.8 = medium effect, >0.8 = large effect.^16^ For the sensitivity analysis, the difference in CHQ scores and effect size between headache and no headache patients was tested with and without primary headache patients. If the exclusion of primary headache patients showed large differences in effect, these patients were excluded from further analysis. Correlation calculations were performed using the Spearman correlation test. Results were corrected for multiple testing.

## RESULTS

### Study Population

In all, 95 parents of patients with sagittal synostosis were approached to participate in the study, of which 68 (71.57%) parents participated in the study by completing the CHQ-PF50. The average age of the participating children was 12.04 years (10.8 to 14.2). Attrition analysis revealed no significant differences between the participating (n = 68) and non-participating (n=27) groups in terms of sex, age at study, age at surgery, and type of surgery (Supplemental Table 1, Supplemental Digital Content 2, http://links.lww.com/SCS/F491).

Of these 68 patients, 30 (44.12%) patients underwent an FBR, and 38 (58.88%) underwent an ESC.

### CHQ-PF50 of Sagittal Synostosis Patients Compared to a Normative Sample

The CHQ-PF50 scales of patients with sagittal synostosis were similar to those of the normal population, with 2 exceptions. CHQ-PF50 showed significantly higher scores in Family cohesion (*P* = 0.02) and lower scores in Mental Health (*P*=0.05) (Supplemental Table 2, Supplemental Digital Content 2, http://links.lww.com/SCS/F491). These items are however of small or medium effect sizes (Supplemental Table 2). In addition, the patient’s age at completing the questionnaire is not correlated with the CHQ scores (Supplemental Table 3, Supplemental Digital Content 2, http://links.lww.com/SCS/F491). The psychosocial summary is slightly lower compared with the norm; however, it is not significant. The physical summary was comparable to the norm.

### The Difference in CHQ-PF50 for the Type of Surgery

There were no differences in CHQ-PF50 items between surgical techniques (Supplemental Table 4, Supplemental Digital Content 2, http://links.lww.com/SCS/F491). The age at surgery is also not correlated to the CHQ scores (Supplemental Table 3, Supplemental Digital Content 2, http://links.lww.com/SCS/F491).

### Headache in Sagittal Synostosis Patient

Out of 69 patients, 48 (70.59%) reported having headache complaints. Nine patients (18.75%) were diagnosed with a primary headache based on the ICHD-3 diagnostic criteria: 3 patients with migraine, 6 patients with tension-type headache, and none with cluster headache. Sensitivity analysis of the subgroup showed no large differences in effect size when excluding primary headache patients from the analysis (Supplemental Digital Content 1, http://links.lww.com/SCS/F490).

Of the 48 patients (70.59%) with headache complaints, 32 patients (66.67%) (of which 16 were FBR and 16 were ESC) reported having headache complaints at least once a month. These patients were categorized as having frequent headaches (Supplemental Table 5, Supplemental Digital Content 2, http://links.lww.com/SCS/F491)

On average, these headaches lasted over 4 hours. Five patients reported additional complaints (eg, poor/blurry sight, nausea, or vomiting) during their headache episodes. A more detailed description of this headache questionnaire is shown in Supplemental Table 6, Supplemental Digital Content 2, http://links.lww.com/SCS/F491.

The CHQ of patients with frequent headache complaints showed a significantly lower score at 9 items.

As one would expect, patients with frequent headache complaints scored worse on bodily pain, with a large effect. Items with medium effect consist of emotional behavior, general behavior, mental health, general health perceptions, parental impact emotional, family activity, physical summary, and psychosocial summary (Supplemental Table 7, Supplemental Digital Content 2, http://links.lww.com/SCS/F491).

**FIGURE 1:**
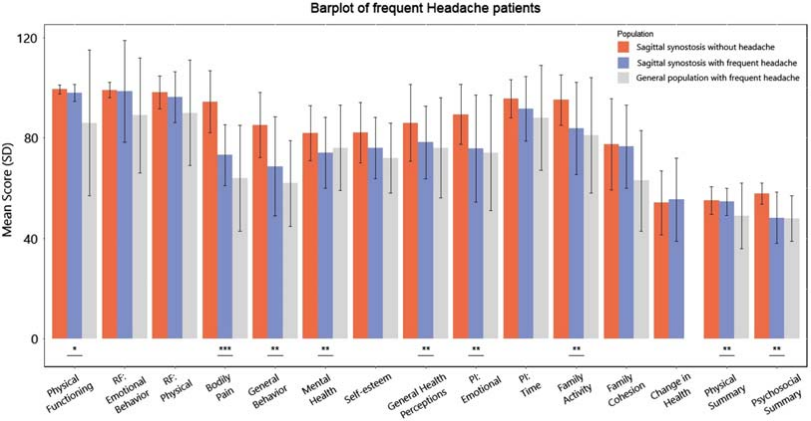
HR-QoL in sagittal synostosis patients with and without frequent headaches, compared with the general population with frequent headaches—*P* < 0.05 between SS with and without frequent headaches. *small effect, **medium effect, ***large effect; RF indicates role functioning; PI, parental impact.

The SS patients with frequent headaches scored higher or similar in all items compared with the general population with frequent headaches (Fig. 1). Due to the unavailability of the original data of Raat et al (2005),^15^ no correct statistical tests could be performed, and differences cannot be analyzed.

Of the 68 patients, one patient encountered ICH upon completing the questionnaire. These patients reported experiencing daily headache complaints. He was diagnosed with ICH before participating in the study. Following the questionnaire, the patient underwent reoperation, which led to a decrease in headache complaints. There were no reported cases of ICH development among any patients after questionnaire completion, as confirmed through follow-up assessments until January 2023.

## DISCUSSION

Sixty-eight parents of patients with treated SS completed the CHQ-PF50, a validated and reliable questionnaire to estimate HR-QoL. Results showed that children with SS had only a similar quality of life, as indicated by the CHQ summary scores, compared with the normal Dutch population; surprisingly, “Family cohesion” was higher, and “Mental Health” was lower in SS patients.

The type of surgery, age at surgery, and age of the patient at CHQ completion did not affect the results.

A few studies have examined the HR-QoL in children with sagittal synostosis.^6,17–21^ Our study is the largest study to date, with a focus solely on sagittal synostosis patients. Other studies tend to have a combined population of various types of craniosynostosis.

Kljajic et al^17^ and Mazzaferro et al^19^ have included separate and detailed analyses of SS patients, whereas the articles by Shavlokhova et al,^18^ Sader et al,^20^ and Cloonan et al^6^ have included SS patients as part of the craniosynostosis group. Kljajic et al^17^ and Mazzaferro et al^19^ found no difference in the HR-QoL between SS patients and the normal population in the majority of the scales, which is in line with our results. In addition, both our study and Kljajic et al^17^ concluded no influence of surgical intervention on HR-QoL.

Children with SS have a higher risk of developing ICH. Since frequent headache complaints are related to papilledema and, therefore, as a possible first symptom of ICH,^10^ one should be aware of headache complaints. The prevalence of frequent headaches in the normal population has a wide range (from 12% to 54%^22–28^). In our study population, 46.06% of the children with SS have frequent headache complaints.

Patients with frequent headaches or primary headaches are known to have a significantly lower HR-QoL in (almost) all domains.^15,29^ This also applies to children with sagittal synostosis. In this study, SS patients with frequent headaches had significantly lower scores of mild to large effects on about 8 CHQ scales, indicating lower HR-QoL due to bodily pain, behavior/emotions, and mental health of the child and parents/family compared with SS patients without headaches. However, in SS patients, frequent headaches had less to no effect on the physical limitation, in contrast to the general population.

### Strengths and Limitations

Some limitations need to be addressed and taken into account when interpreting the results.

First of all, with the use of a questionnaire, the possibility of selection bias arises. The high response rate (71.57%) and the attrition analysis however minimize the risk of selection bias. Even though it might be limited, a larger study sample would still increase the probability of the assumptions in this study. The response rate in our study conforms to the suggested minimally required response rate of at least 60% by Livingston et al (2012).^30^

Besides the potential selection bias, a more common limitation of surveys is the risk of recall bias. Both the CHQ and the detailed headache questionnaire are self-administered surveys and are prone to inaccurate or incomplete recollections of events by the respondents (in this case, the parents). Past literature^31^ has concluded that pain complaints are evaluated more negatively on a retrospective questionnaire compared with a prospective diary. Indicating a possible overestimation of the severity of pain in our questionnaire. In addition, the headache questionnaire is served as a diagnostic tool and therefore does not include questions regarding experience.

Specifically regarding the use of the CHQ-PF50, there are also some limitations. This study used a validated and translated questionnaire to assess the HR-QoL. The questionnaire is designed for parents of children between the ages of 5 and 18 years. However, the normative sample is based on a population between 5 and 13 years old.^11^ Even though we found no correlation between the age of completing the questionnaire and the scores, the population between 14 and 18 cannot be accurately compared.

In addition, the questionnaire’s perspective is limited to that of the parents since the perspective of the child is not included. Kljajic et al^17^ found a difference in perspective between parents and children in physical function and school function. School function in the PedsQL evolves around schoolwork and is equivalent to Role functioning: emotional behavior and physical in the CHQ-PF50. Physical functioning is present in both the PedsQL and the CHQ-PF50. These scores can be overestimated in our study population as the child’s perspective is not considered.

## CONCLUSION

In general, children who are treated for SS have a relatively normal HR-QoL based on parents’ perspective; the impact on HR-QoL is relatively small. In family cohesion and mental health, children with sagittal synostosis differ mildly from the normal population. The type of surgery, age at surgery, and age at CHQ completion did not affect the results. In all, 47.06% of the patients have frequent headache complaints, which has a negative effect on the HR-QoL in both SS patients and the normal population. The CHQ-PF50 will give insight into a patient’s HR-QoL and can help identify which patients require a psychological evaluation and/or guidance.

## Supplementary Material

SUPPLEMENTARY MATERIAL

## References

[1] Mathijssen IMJ . Working Group Guideline C. Updated Guideline on Treatment and Management of Craniosynostosis. J Craniofac Surg 2021;32:371–450 33156164 10.1097/SCS.0000000000007035 PMC7769187

[2] van Veelen MC Jippes M Carolina JA . Volume measurements on three-dimensional photogrammetry after extended strip versus total cranial remodeling for sagittal synostosis: A comparative cohort study. J Craniomaxillofac Surg 2016;44:1713–1718 27591089 10.1016/j.jcms.2016.07.029

[3] Frostell A Haghighi M Bartek J Jr . Improved cephalic index following early cranial vault remodeling in patients with isolated nonsyndromic sagittal synostosis. Neurosurg Focus 2021;50:E7 10.3171/2021.1.FOCUS201017 33794490

[4] Al-Shaqsi SZ Rai A Forrest C . Standardization of cranial index measurement in sagittal craniosynostosis. J Craniofac Surg 2019;30:366–369 30531285 10.1097/SCS.0000000000005034

[5] Fischer S Maltese G Tarnow P . Intracranial volume is normal in infants with sagittal synostosis. J Plast Surg Hand Surg 2015;49:62–64 25363060 10.3109/2000656X.2014.971804

[6] Cloonan YK Collett B Speltz ML . Psychosocial outcomes in children with and without non-syndromic craniosynostosis: findings from two studies. Cleft Palate Craniofac J 2013;50:406–413 22315944 10.1597/11-074 PMC3475758

[7] Peter M Fayers DM . Quality of Life: The Assessment, Analysis and Reporting of Patient-reported Outcomes. Chichester: Wiley Blacwell; 2016

[8] Raposo-Amaral CE Neto JGJ Denadai R . Patient-reported quality of life in highest-functioning Apert and Crouzon syndromes: a comparative study. Plast Reconstr Surg 2014;133:182e–191ee 10.1097/01.prs.0000437260.31693.75 24469189

[9] Raposo-Amaral CE Raposo-Amaral CA Garcia Neto JJ . Apert syndrome: quality of life and challenges of a management protocol in Brazil. J Craniofac Surg 2012;23:1104–1108 22777480 10.1097/SCS.0b013e318258814a

[10] van de Beeten SDC Mathijssen IMJ Kamst NW . Headache in postoperative isolated sagittal synostosis. Plast Reconstr Surg 2019;143:798e–805e 10.1097/PRS.0000000000005481 30921138

[11] Raat H Bonsel GJ Essink-Bot ML . Reliability and validity of comprehensive health status measures in children: The Child Health Questionnaire in relation to the Health Utilities Index. J Clin Epidemiol 2002;55:67–76 11781124 10.1016/s0895-4356(01)00411-5

[12] Ware JE Jr Gandek B Kosinski M . The equivalence of SF-36 summary health scores estimated using standard and country-specific algorithms in 10 countries: results from the IQOLA Project. International Quality of Life Assessment. J Clin Epidemiol 1998;51:1167–1170 9817134 10.1016/s0895-4356(98)00108-5

[13] Landgraf JM . Child health questionnaire (CHQ). Encyclopedia of quality of life and well-being research. Springer; 2020. p 1–6

[14] Genootschap NH Hoofdpijn. 2021.

[15] Raat H Botterweck AM Landgraf JM . Reliability and validity of the short form of the child health questionnaire for parents (CHQ-PF28) in large random school based and general population samples. J Epidemiol Community Health 2005;59:75–82 15598731 10.1136/jech.2003.012914 PMC1763365

[16] Cohen J . Statistical power analysis for the behavioral sciences. Routledge; 1988

[17] Kljajic M Maltese G Tarnow P . Health-related quality of life of children treated for non-syndromic craniosynostosis. J Plast Surg Hand Surg 2022:1–7 36409664 10.1080/2000656X.2022.2147532

[18] Shavlokhova V Gruninger S Hoffmann J . Health-related quality of life in children after surgical treatment of non-syndromal craniosynostosis. J Craniomaxillofac Surg 2021;49:655–658 34366005 10.1016/j.jcms.2019.04.007

[19] Mazzaferro DM Naran S Wes AM . Quality of life in adults with nonsyndromic craniosynostosis. Plast Reconstr Surg 2018;141:1474–1482 29579020 10.1097/PRS.0000000000004408

[20] Sader N Mehta V Hart S . Quality of life and satisfaction in surgical versus conservative treatment of nonsyndromic children with craniosynostosis. J Neurosurg Pediatr 2022;29:60–65 34653978 10.3171/2021.5.PEDS2136

[21] Boltshauser E Ludwig S Dietrich F . Sagittal craniosynostosis: cognitive development, behaviour, and quality of life in unoperated children. Neuropediatrics 2003;34:293–300 14681754 10.1055/s-2003-44667

[22] Fendrich K Vennemann M Pfaffenrath V . Headache prevalence among adolescents--the German DMKG headache study. Cephalalgia 2007;27:347–354 17376112 10.1111/j.1468-2982.2007.01289.x

[23] Laimi K Metsahonkala L Anttila P . Outcome of headache frequency in adolescence. Cephalalgia 2006;26:604–612 16674770 10.1111/j.1468-2982.2004.01084.x

[24] Petersen S Bergstrom E Brulin C . High prevalence of tiredness and pain in young schoolchildren. Scand J Public Health 2003;31:367–374 14555373 10.1080/14034940210165064

[25] Swain MS Henschke N Kamper SJ . An international survey of pain in adolescents. BMC Public Health 2014;14:447 24885027 10.1186/1471-2458-14-447 PMC4046513

[26] Zwart JA Dyb G Holmen TL . The prevalence of migraine and tension-type headaches among adolescents in Norway. The Nord-Trondelag Health Study (Head-HUNT-Youth), a large population-based epidemiological study. Cephalalgia 2004;24:373–379 15096226 10.1111/j.1468-2982.2004.00680.x

[27] Szperka C . Headache in children and adolescents. Continuum (Minneap Minn) 2021;27:703–731 34048400 10.1212/CON.0000000000000993 PMC9455826

[28] Nieswand V Richter M Gossrau G . Epidemiology of headache in children and adolescents-another type of pandemia. Curr Pain Headache Rep 2020;24:62 32840694 10.1007/s11916-020-00892-6 PMC7447651

[29] Bruijn J Arts WF Duivenvoorden H . Quality of life in children with primary headache in a general hospital. Cephalalgia 2009;29:624–630 19175611 10.1111/j.1468-2982.2008.01774.x

[30] Livingston EH Wislar JS . Minimum response rates for survey research. Arch Surg 2012;147:110 22351903 10.1001/archsurg.2011.2169

[31] van den Brink M Bandell-Hoekstra EN Abu-Saad HH . The occurrence of recall bias in pediatric headache: a comparison of questionnaire and diary data. Headache 2001;41:11–20 11168599 10.1046/j.1526-4610.2001.111006011.x

