# Supplementary material for: Health-related Quality of Life in Children and Adolescents With Sagittal Synostosis
Source: J Craniofac Surg. 2023 Sep 8;34(8):2284–7. doi: 10.1097/SCS.0000000000009733 (PMC10597426; doi:10.1097/SCS.0000000000009733)
Supplement: SUPPLEMENTARY MATERIAL [file scs-34-2284-s001.docx]

|  | **Total cohort (N = 68)** | | | | **Without primary headache patients (68 – 9; n = 59)** | | | |
| --- | --- | --- | --- | --- | --- | --- | --- | --- |
| **CHQ Scales** | Without Frequent headache  Mean (SD)  N=36 | With frequent Headache  Mean (SD)  N=32 | P value ^1^ | Effect size R ^2^ | Without Frequent headache  Mean (SD)  N=34 | With frequent Headache  Mean (SD)  N=25 | P value ^1^ | Effect size R ^2^ |
| Physical functioning (PF) | 99.38 (1.77) | 97.92 (3.37) | 0.063 | 0.227 | 99.3 (1.82) | 97.8 (3.59) | **0.049** | 0.256 |
| Role functioning: Emotional.behaviour (REB) | 99.07 (3.11) | 89.58 (20.34) | **0.011** | **0.309** | 99.0 (3.20) | 90.2 (20.4) | **0.017** | 0.**312** |
| Role functioning: Physical (RP) | 98.15 (6.64) | 96.35 (10.14) | 0.5445 | 0.075 | 98.0 (6.82) | 97.3 (9.23) | 0.975 | 0.006 |
| Bodily pain (BP) | 94.44 (12.29) | 73.13 (12.29) | **<0.001** | **0.577** | 94.7 (12.4) | 73.2 (19.7) | **<0.001** | **0.595** |
| General behavior (GB) | 85.12 (13.05) | 68.59 (19.75) | **<0.001** | **0.458** | 84.9 (13.4) | 68.5 (19.1) | **<0.001** | **0.435** |
| Mental health (MH) | 81.94 (11.04) | 74.06 (14.22) | **0.025** | 0.273 | 82.2 (11.2) | 73.2 (13.9) | **0.014** | 0.**332** |
| Self-esteem (SE) | 82.06 (11.99) | 76.04 (12.25) | 0.061 | 0.227 | 81.7 (12.1) | 77.3 (10.8) | 0.181 | 0.176 |
| General health perceptions (GH) | 85.97 (15.32) | 78.20 (14.44) | **0.015** | 0.296 | 85.7 (15.7) | 76.1 (15.1) | **0.010** | 0.**336** |
| Parental impact: Emotional (PE) | 89.35 (11.89) | 75.78 (21.41) | **0.006** | **0.335** | 89.7 (12.1) | 75.0 (22.4) | **0.007** | 0.**354** |
| Parental impact: Time (PT) | 95.68 (7.6) | 91.67 (12.93) | 0.326 | 0.120 | 95.4 (7.8) | 92.0 (13.4) | 0.662 | 0.058 |
| Family activity (FA) | 95.14 (9.99) | 83.72 (18.37) | **<0.001** | **0.400** | 95 (10.2) | 82.8 (19.6) | **<0.001** | **0.400** |
| Family cohesion (FC) | 77.50 (18.22) | 76.56 (16.58) | 0.693 | 0.049 | 76.6 (18.3) | 77.2 (17.6) | 0.954 | 0.009 |
| Change in health (CH) | 54.17 (12.68) | 55.47 (16.48) | 0.635 | -0.059 | 54.4 (13.0) | 54.0 (11.8) | 0.773 | -0.046 |
| Physicial summary (PHS) | 57.74 (4.20) | 54.54 (5.43) | **0.007** | **0.330** | 57.74 (4.31) | 54.3 (5.10) | **0.002** | 0.**389** |
| Psychosocial summary (PSS) | 54.99 (5.46) | 48.33 (10.19) | **0.004** | **0.347** | 55.0 (5.6) | 48.4 (9.98) | **0.007** | 0.**348** |

**Supplemental Digital Content 1:** Subgroup Sensitivity analysis of patients with primary headache
