# Supplementary material for: Health-related Quality of Life in Children and Adolescents With Sagittal Synostosis
Source: J Craniofac Surg. 2023 Sep 8;34(8):2284–7. doi: 10.1097/SCS.0000000000009733 (PMC10597426; doi:10.1097/SCS.0000000000009733)
Supplement: SUPPLEMENTARY MATERIAL [file scs-34-2284-s002.docx]

| **Parameters** | **Participating**  **N = 68** | **Non participating**  **N = 27** |  |
| --- | --- | --- | --- |
| Sex - N (%) |  |  | 0.422 |
| Male | 57 (83.8%) | 20 (74.1%) |  |
| Female | 11 (16.2%) | 7 (25.9%) |  |
| Headache (at least once a month) – N (%) |  |  |  |
| Yes | 32 | - |  |
| No | 36 | - |  |
| Type of surgery - N (%) |  |  | 0.269 |
| FBR | 30 (44.1%) | 16 (59.3%) |  |
| ESC | 38 (55.9%) | 11 (40.7%) |  |
| Age at surgery - Median (IQR) |  |  | 0.18 |
| Total | 0.51 (0.37-0.89) | 0.800 (0.39 – 1.06) |  |
| FBR | 0.93 (0.82-1.0) | 0.960 (0.85 – 1.12) |  |
| ESC | 0.38 (0.34-0.44) | 0.360 (0.33 – 0.41) |  |
| Age at survey – Median (IQR) |  |  |  |
| Total | 12.04 (10.8-14.2) |  |  |
| FBR | 12.96 (11.9-17.4) |  |  |
| ESC | 11.03 (9.8-13.1) |  |  |

Supplemental Table 1. General characteristics of the patients population and attrition analysis .

FBR, fronto-biparietal remodeling; ESC, extended strip craniotomy

| **CHQ Scales** | **Baseline Score mean, (SD)**  **N=68** | **Normative sample mean, (SD)  N= 353** | **p value** | **Cohen’s d** |
| --- | --- | --- | --- | --- |
| Physical functioning (PF) | 98.7 (2.9) | 99.1 (4.3) | 0.25 | -0.14 |
| Role functioning: Emotional.behaviour (REB) | 94.6 (14.8) | 97.9 (7.2) | 0.07 | -0.22 |
| Role functioning: Physical (RP) | 97.3 (8.5) | 95.8 (15.6) | 0.15 | 0.18 |
| Bodily pain (BP) | 84.4 (19.3) | 85.7 (17.2) | 0.59 | -0.07 |
| General behavior (GB) | 77.3 (17.9) | 78.5 (13.1) | 0.60 | -0.06 |
| Mental health (MH) | 78.2 (13.2) | 81.4 (12.1) | **0.05** | -0.24 |
| Self-esteem (SE) | 79.2 (12.4) | 79.2 (11.0) | 0.99 | 0 |
| General health perceptions (GH) | 82.3 (15.3) | 82.9 (13.4) | 0.75 | -0.04 |
| Parental impact: Emotional (PE) | 83.0 (18.2) | 86.3 (15.2) | 0.14 | -0.18 |
| Parental impact: Time (PT) | 93.8 (10.6) | 94.0 (13.0) | 0.87 | -0.02 |
| Family activity (FA) | 89.8 (15.5) | 91.5 (11.9) | 0.36 | -0.11 |
| Family cohesion (FC) | 77.1 (17.3) | 72.2 (19.4) | **0.02** | 0.28 |
| Change in health (CH) | 54.8 (14.5) | 56.1 (18.4) | 0.46 | -0.09 |
| Physicial summary (PHS) | 56.3 (5.0) | 56.4 (5.7) | 0.88 | 0.03 |
| Psychosocial summary (PSS) | 51.9 (8.7) | 53.2 (6.4) | 0.20 | 0.16 |

**Supplemental** **Table 2 CHQ-PF50: Children with operated sagittal synostosis compared with healthy children, and with children with primary headache or with syndromic craniosynostosis.**
* Significant difference (p<0.05; two-sided) measured by one-sample t-test.
NA, not available.

| **CHQ Scales** | **Age at CHQ (r)** | **Age at surgery (r)** |
| --- | --- | --- |
| Physical functioning (PF) | -0.10 (-0.33; 0.14) | 0.02 (-0.22; 0.26) |
| Role functioning: Emotional.behaviour (REB) | 0.18 (-0.06; 0.40) | 0.03 (-0.21; 0.26) |
| Role functioning: Physical (RP) | -0.14 (-0.37; 0.10) | 0.07 (-0.17; 0.30) |
| Bodily pain (BP) | 0.07 (-0.17; 0.30) | -0.03 (-0.27; 0.21) |
| General behavior (GB) | 0.29 (0.05; 0.49) | 0.15 (-0.09, 0.38) |
| Mental health (MH) | 0.26 (0.02; 0.47) | 0.21 (-0.03; 0.42) |
| Self-esteem (SE) | -0.07 (-0.31; 0.17) | 0.00 (-0.24, 0.24) |
| General health perceptions (GH) | 0.11 (-0.13; 0.34) | -0.03 (-0.26; 0.21) |
| Parental impact: Emotional (PE) | 0,17 (-0.07; 0.39) | 0.13 (-0.11;0.36) |
| Parental impact: Time (PT) | 0.11 (-0.13; 0.34) | 0.09 (-0.15; 0.32) |
| Family activity (FA) | 0.06 (-0.18; 0.29) | -0.02 (0.25; 0.22) |
| Family cohesion (FC) | 0.07 (0.17; 0.30) | -0.03 (-0.27; 0.21) |
| Change in health (CH) | 0.10 (-0.14; 0.33) | 0.30 (0.07; 0.50) |
| Physicial summary (PHS) | -0.05 (-0.29; 0.19) | -0.05 (-0.29; 0.19) |
| Psychosocial summary (PSS) | 0.23 (-0.01, 0.44) | 0.15 (-0.09; 0.37) |

**Supplemental** **Table 3 correlation between CHQ and age at time of the questionnaire, and age at surgery**

| **CHQ Scales** | **FBR (n = 30)** | **ESC (N = 38)** | **P value ^1^** | **Effect size R ^2^** |
| --- | --- | --- | --- | --- |
| Physical functioning (PF) | 98.89 (2.26) | 98.53 (3.34) | 1.000 | 0.000 |
| Role functioning: Emotional.behaviour (REB) | 95.19 (11.92) | 94.15 (16.89) | 0.907 | -0.015 |
| Role functioning: Physical (RP) | 97.78 (7.24) | 96.93 (9.37) | 0.907 | 0.016 |
| Bodily pain (BP) | 83.33 (19.18) | 85.26 (19.69) | 0.571 | -0.070 |
| General behavior (GB) | 81.36 (15.06) | 74.17 (20.11) | 0.196 | 0.158 |
| Mental health (MH) | 80.50 (11.92) | 76.45 (13.95) | 0.258 | 0.138 |
| Self-esteem (SE) | 78.19 (10.76) | 80.04 (13.64) | 0.714 | -0.045 |
| General health perceptions (GH) | 83.11 (13.15) | 81.69 (16.97) | 0.960 | 0.007 |
| Parental impact: Emotional (PE) | 85.00 (19.25) | 81.36 (17.48) | 0.195 | 0.158 |
| Parental impact: Time (PT) | 95.19 (9.08) | 92.69 (11.63) | 0.434 | 0.096 |
| Family activity (FA) | 89.58 (16.91) | 89.91 (14.58) | 0.938 | 0.010 |
| Family cohesion (FC) | 77.17 (16.06) | 76.97 (18.51) | 0.888 | -0.018 |
| Change in health (CH) | 59.17 (17.96) | 51.32 (9.97) | 0.062 | 0.227 |
| Physicial summary (PHS) | 56.07 (4.35) | 56.35 (5.58) | 0.533 | -0.076 |
| Psychosocial summary (PSS) | 53.13 (7.55) | 50.85 (9.42) | 0.229 | 0.147 |

**Supplemental** **Table 4 CHQ-PF50 between surgical techniques**

1) Mann Whitney test

2) Effect Size in r; <0.3 = small effect, 0.3 – 0.5 = medium effect, >0.5 = large effect

FBR, fronto-biparietal remodeling; ESC, extended strip craniotomy

|  | **N = 68** | **FBR (n = 30)** | **ESC (n = 38)** |
| --- | --- | --- | --- |
| Headache |  |  |  |
| Yes | 48 (70.59%) | 21 (70.0%) | 27 (71.05%) |
| No | 20 (29.41%) | 9 (30.0%) | 11 (28.95%) |
| Primary headache diagnosis |  |  |  |
| Migraine | 3 (4.41%) | 1 (3.33%) | 2 (5.26%) |
| Tension type headache | 6 (8.82%) | 3 (10.0%) | 3 (7.89%) |
| Cluster headache | 0 | 0 | 0 |
| Frequency | **N = 48** | **N = 21** | **N = 27** |
| Daily | 2 (4.17%) | 1 (4.76%) | 1 (3.70%) |
| Weekly | 11 (22.92%) | 7 (33.33%) | 4 (14.81%) |
| Monthly | 19 (39.58%) | 8 (38.10%) | 11 (40.74%) |
| Once every 3 months | 9 (18.75%) | 3 (14.29%) | 6 (22.22%) |
| Once every 6 months | 7 (14.58%) | 2 (9.52%) | 5 (18.82%) |

**Supplemental** **Table 5 Headache frequency and Primary headache**

FBR, fronto-biparietal remodeling; ESC, extended strip craniotomy

|  | **All headache (n = 32)** | **FBR (N = 16)** | **ESC (N = 16)** |
| --- | --- | --- | --- |
| How long does the headache episode take (min) |  |  |  |
| Mean | 275.2 | 452.2 | 98.3 |
| Median | 60 | 90 | 45 |
| IQR | 25-180 | 30 - 465 | 8 – 97.5 |
| At what time of the day* |  |  |  |
| Morning | 5 (15.63%) | 2 (12.50%) | 3 (18.75%) |
| Night | 3 (9.38%) | 1 (6.25%) | 2 (12.50%) |
| Midday | 7 (21.87%) | 4 (25.0%) | 3 (18.75%) |
| After school | 11 (34.38%) | 4 (25.0%) | 7 (43.85%) |
| After exercise | 2 (6.25%) | 1 (6.25%) | 1 (6.25%) |
| Variable/no pattern | 10 (31.25%) | 4 (25.0%) | 6 (37.50%) |
| At what day of the week |  |  |  |
| Only Monday – Friday | 13 (40.63%) | 8 (50.0%) | 5 (31.25%) |
| Only Weekend |  |  |  |
| Both | 19 (59.38%) | 8 (50.0%) | 11 (68.75%) |
| Do you wake up with headache |  |  |  |
| Yes | 4 (12.50%) | 2 (12.50%) | 2 (12.50%) |
| No | 28 (87.50%) | 14 (87.50%) | 14 (87.50%) |
| Other complaints during headache episodes |  |  |  |
| Poor/burry sight | 1 (3.13%) | 1 (6.25%) |  |
| Nausea | 4 (12.50%) | 2 (12.50%) | 2 (12.50%) |
| Vomitting | 1 (3.13 %) |  | 1 (6.25%) |
| Watery eyes |  |  |  |
| Running nose |  |  |  |
| Red eyes/Ptosis |  |  |  |
| Fatigue |  |  |  |
| No complains | 26 (81.25%) | 13 (81.25%) | 13 (81.25%) |
| Location headache* |  |  |  |
| Frontal | 21 (65.63%) | 12 (75.00%) | 9 (56.25%) |
| Occipital | 3 (9.38%) | 1 (6.25%) | 2 (12.50%) |
| Sides | 4 (12.50%) | 0 | 4 (25.00%) |
| Neck | 3 (9.38%) | 0 | 3 (18.75%) |
| around the eyes | 2 (6.25%) | 1 (6.25%) | 1 (6.25%) |
| At scar | 4 (12.50%) | 2 (12.50%) | 2 (12.50%) |
| All sides | 0 |  |  |
| Undefined | 2 (6.25%) |  |  |
| Headache one side/both sides |  |  |  |
| One side | 17 (53.13%) | 8 (50.0%) | 6 (37.50%) |
| Both sides | 14 (43.75%) | 8 (50.0%) | 9 (56.25%) |
| NA | 1 (3.13%) | 0 | 1 (6.25%) |
| Headache originates from the neck |  |  |  |
| Yes | 3 (9.38%) | 0 | 3 (18.75%) |
| No | 28 (87.50%) | 16 (100%) | 12 (75.0%) |
| NA | 1 (3.13%) |  | 1 (6.25%) |
| Pain |  |  |  |
| Lingering pain | 12 (37.50%) | 5 (31.25%) | 7 (43.75%) |
| Oppressive pain | 10 (31.25%) | 7 (43.85%) | 3 (18.75%) |
| Sharp pain | 5 (15.63%) | 2 (12.50%) | 3 (18.75%) |
| Dull pain | 2 (6.25%) | 2 (12.50%) | 0 |
| Constant pain | 1 (3.13%) | 0 | 1 (6.25%) |
| NA | 2 (6.25%) | 0 | 2 (12.50%) |
| Migraine in the Family |  |  |  |
| Yes | 19 (59.38%) | 12 (75.0%) | 7 (43.75%) |
| No | 11 (34.38%) | 4 (25.0%) | 7 (43.75%) |
| NA | 2 (6.25%) |  | 2 (12.50%) |

**Supplemental** **Table 6 Headache Questionnaire**

* Multiple answers possible

FBR, fronto-biparietal remodeling; ESC, extended strip craniotomy

| **CHQ Scales** | **Without Frequent headache  Mean (SD)**  **N=36** | **With frequent Headache  Mean (SD)  N=32** | **P value ^1^** | **Effect size R ^2^** |
| --- | --- | --- | --- | --- |
| Physical functioning (PF) | 99.38 (1.77) | 97.92 (3.37) | 0.063 | 0.227 |
| Role functioning: Emotional.behaviour (REB) | 99.07 (3.11) | 89.58 (20.34) | **0.011** | 0.309 |
| Role functioning: Physical (RP) | 98.15 (6.64) | 96.35 (10.14) | 0.5445 | 0.075 |
| Bodily pain (BP) | 94.44 (12.29) | 73.13 (12.29) | **<0.001** | **0.577** |
| General behavior (GB) | 85.12 (13.05) | 68.59 (19.75) | **<0.001** | 0.458 |
| Mental health (MH) | 81.94 (11.04) | 74.06 (14.22) | **0.025** | 0.273 |
| Self-esteem (SE) | 82.06 (11.99) | 76.04 (12.25) | 0.061 | 0.227 |
| General health perceptions (GH) | 85.97 (15.32) | 78.20 (14.44) | **0.015** | 0.296 |
| Parental impact: Emotional (PE) | 89.35 (11.89) | 75.78 (21.41) | **0.006** | 0.335 |
| Parental impact: Time (PT) | 95.68 (7.60) | 91.67 (12.93) | 0.326 | 0.120 |
| Family activity (FA) | 95.14 (9.99) | 83.72 (18.37) | **<0.001** | 0.400 |
| Family cohesion (FC) | 77.50 (18.22) | 76.56 (16.58) | 0.693 | 0.049 |
| Change in health (CH) | 54.17 (12.68) | 55.47 (16.48) | 0.635 | -0.059 |
| Physicial summary (PHS) | 57.74 (4.20) | 54.54 (5.43) | **0.007** | 0.330 |
| Psychosocial summary (PSS) | 54.99 (5.46) | 48.33 (10.19) | **0.004** | 0.347 |

**Supplemental** **Table 7 CHQ-PF50 in SS patients with and without frequent headache**

1) Mann Whitney test

2) Effect Size in r; <0.3 = small effect, 0.3 – 0.5 = medium effect, >0.5 = large effect
